# Additively manufactured, solid object structures for adjustable image contrast in Magnetic Resonance Imaging

**DOI:** 10.1016/j.zemedi.2022.03.003

**Published:** 2022-05-18

**Authors:** Alejandra Valladares, Gunpreet Oberoi, Andreas Berg, Thomas Beyer, Ewald Unger, Ivo Rausch

**Affiliations:** a QIMP Team, Centre for Medical Physics and Biomedical Engineering, Medical University of Vienna, Vienna, Austria; b Centre for Medical Physics and Biomedical Engineering, Medical University of Vienna, Vienna, Austria; c Centre for Medical Physics and Biomedical Engineering, MR-Physics, Medical University of Vienna, Vienna, Austria; d High-field MR-Center, Medical University of Vienna, Vienna, Austria

**Keywords:** MRI, Physical phantoms, Tumour heterogeneity, Additive manufacturing, PolyJet, 3D printing, STL, Standard tessellation language, AM, Additive manufacturing, CAD, Computer-aided design, GRE, Gradient-echo, ROI, Region-of-interest, SD, Standard deviation, SE, Spin-echo, SNR, Signal-to-noise-ratio, TE, Echo time, TR, Repetition time

## Abstract

The choice of materials challenges the development of Magnetic Resonance Imaging (MRI) phantoms and, to date, is mainly limited to water-filled compartments or gel-based components. Recently, solid materials have been introduced through additive manufacturing (AM) to mimic complex geometrical structures. Nonetheless, no such manufactured solid materials are available with controllable MRI contrast to mimic organ substructures or lesion heterogeneities. Here, we present a novel AM design that allows MRI contrast manipulation by varying the partial volume contribution to a ROI/voxel of MRI-visible material within an imaging object. Two sets of 11 cubes and three replicates of a spherical tumour model were designed and printed using AM. Most samples presented varying MRI-contrast in standard MRI sequences, based mainly on spin density and partial volume signal variation. A smooth and continuous MRI-contrast gradient could be generated in a single-compartment tumour model. This concept supports the development of more complex MRI phantoms that mimic the appearance of heterogeneous tumour tissues*.*

## 1 Introduction

Magnetic Resonance Imaging (MRI) is a well-established and widely used non-invasive tomographic imaging technique. It provides excellent soft-tissue contrast with high spatial resolution and functional and metabolic information of structures and pathologies [1]. In oncology, MRI plays an essential role in detecting and characterising malignancies. In particular, its excellent soft-tissue contrast and the availability of several imaging sequences allow for the visualisation of tumour heterogeneity, thus enabling improved diagnosis, treatment planning, and follow-up [2].

In recent years, the assessment of tumour heterogeneity through radiomics combined with machine learning (ML) and artificial intelligence (AI) has shown potential for better disease characterisation and treatment outcome prediction [3], [4], [5], [6]. As such, radiomics may become an integral part of personalised medicine approaches [5]. Radiomics-based ML and AI approaches rely on large amounts of imaging data and standardised imaging protocols to address confounding factors, such as data variability [7]. However, particularly in MRI, non-standardised imaging protocols are commonplace and limited comparability of image data between imaging centres and MRI systems are roadblocks in the development, validation and broad implementation of radiomics-based approaches [8], [9]. Here, reference imaging objects (a.k.a. phantoms) serve as tools for developing and validating standardised and harmonised image acquisition protocols, image processing, and image analysis methods.

Unlike Computerized Tomography (CT), where solids with different densities are used as phantom materials, MRI phantoms mainly comprise of multi-compartment models filled with different aqueous solutions or gel-type materials since solids do not cause signals from using standard MRI sequences [10]. Although aqueous and gel-based models are suitable for specific applications, their reproducibility and temporal stability are limited. For example, agarose gel phantoms require specific preparation and storage conditions to maintain their MRI properties over time [11]. Furthermore, texture in MR images depends on several imaging parameters, such as slice thickness, voxel size, field of view and field strength [4], [12]. Therefore, in the context of characterising tumour heterogeneities, test objects for adequate calibration and quality control of quantitative MRI may demand a tailored adjustment of the test object design to the respective imaging situation.

Over the last decade, additive manufacturing (AM), also known as 3D printing technology, has been increasingly used to design and construct physical phantoms in MRI [13], [14], [15]. It is usually used to create compartments that are subsequently filled with solutions or gels with different added contrast agents yielding the desired MRI contrast [16], [17], [18], [19], [20]. However, AM of phantom compartments encompasses similar challenges to any other liquid or gel-fillable phantoms regarding preparation and handling [11]. Soft silicone-like materials obtained by curing methods reported visibility and MRI properties similar to some organ tissues, such as liver, muscle, white matter and others [21] but demand specific printing materials adjusted to the particular envisaged MRI contrast. Few 3D printed and other solid materials are visible using ultrashort echo-time (UTE) pulse sequence. These are used for the recreation of bone and dental tissues, as reported, e.g. in [22], [23], [24].

Lately, a commercially available material, RGD525 (Stratasys Ltd., EdenPrairie, MN, USA), has been reported with MRI-signal generating properties and, thus, used to create MRI phantoms for image-guided therapy simulation [13], quality control of MRI radiomic features [14] and direct attenuation correction in PET/MRI [15]. To our knowledge, there is no report on a solid material capable of reproducing variable MRI-contrast in tumour heterogeneities at sufficient quality.

In this work, we propose a novel design technique for polyjet AM to build solid objects with variable contrast in MRI, based mainly on spin density, by taking advantage of the MRI-signal generating properties of a commonly used 3D printing support material. Furthermore, we demonstrate how the generated MRI-contrast can be adjusted by this technique and present an application for mimicking tumour heterogeneity.

## 2 Materials and methods

### 2.1 Polyjet technique

PolyJet^TM^ (Stratasys Ltd., Eden Prairie, MN, USA) is an AM technique that offers multi-material printing of objects in a wide range of polymers, ranging from flexible to rigid [25], [26]. It allows printing 3D objects with a precision of 600 × 600 dots per inch (dpi) in the XY plane, providing a minimal size of a deposited material dot of approx. 0.043 mm and 16–30 μm layer thickness in the Z direction [27], [28]. A polyjet printer simultaneously jets a specialised gel-like carrier material, mainly composed of acrylic acid and 2-hydroxyethyl ester (1–3%), to support complex features of a design [26]. After printing, this material is intended to be removed manually (waterjet cleaning) or by chemical cleaning (immersion in sodium hypochlorite). Within this work, we used a new design technique to utilise this support material to achieve a controllable and variable MRI contrast within a solid, 3D printed object.

### 2.2 Object design

**Figure 1:**
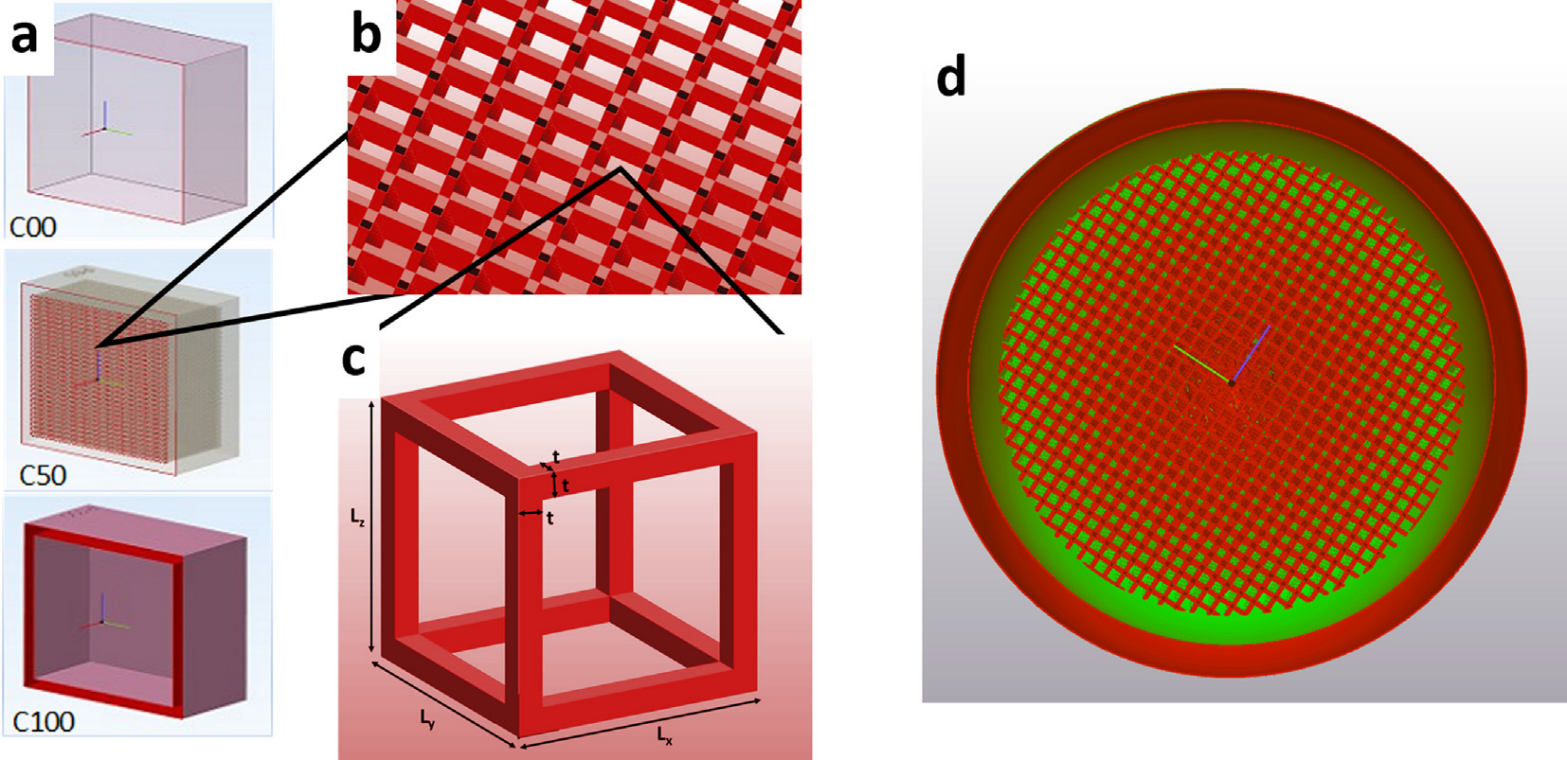
CAD models of the samples. (a) Internal structure: model of the C00 (C% = 0%), C50 (C% = 50%) and C100 (C% = 100%) cubic test object. (b) Magnified example of the inner building material structure. (c) Example of an inner basic cube building up the internal structure with a fixed outer dimension of 1 × 1 × 1 mm^3^ and a square pillar edge length of t. The macroscopic concentration of the MR visible support material is controlled by varying t. (d) Schematic drawing of the tumour model where t was varied from the centre of the sphere (t = max, no support material, 100% building material – red colour) radially towards the outside where t becomes t = 0 (100% support material – green colour).

The general object design was based on reconstructing the desired geometry using Materialize 3-Matic 13.0 software (Materialise, Leuven, Belgium). The variation of the MRI signal was realised by varying the macroscopic volumetric concentration of the support material (C%) within a matrix of the building material. The matrix of the building material was constructed as a three-dimensional grid of cubic structures (Fig. 1 ,b); each single cube consisted of squared pillars at the edges of a virtual cube (Fig. 1, c). The outer dimension of each cube (L) was fixed to *L* = 1 mm in all directions (*L* = *L*_x_ = *L*_y_ = *L*_z_ = 1 mm). C% was varied by adjusting the edge length of the squared pillars (t) (Fig. 1, c) towards the inside of the cube, thus reducing the volume for the support material. A 2 mm thick enclosing structure consisting of solid building material was added at the outside of the object to keep everything encapsulated.

To test the described design and manufacturing technique, eleven cubic samples of Volume *V* = 34 × 34 × 34 mm^3^ were designed with support material contents of C% = 0% (solid building material) to C% = 100% (pure support material) in steps of 10% points.

Three tumour models representing a necrotic tumour were designed to assess the ability to vary MRI visibility within the objects. The CAD design of the tumour model was based on a sphere with an outer diameter *d* = 38 mm. The inner matrix structure of the sphere was similar to that for the cubic samples. However, the support material concentration was quasi-continuously (in 1 mm steps) adjusted from C% = 0% in the center to C% = 100% at the outside. This was done by decreasing the edge length t of the squared pillars building up the basic 1 mm^3^ cubic inner structure from the maximum size *t* = *L*/2 (*C* = 0%) to *t* = 0 (*C* = 100%) from the centre of the sphere towards the outside (Fig. 1.d)

### 2.3 Additively manufactured phantom

The CAD models were translated to the printer in a printable Standard Tessellation Language (STL) format. First, the STLs of the 11 cubic samples (C00–C100) were transferred to the PolyJet^TM^ printer Connex3 Objet500 (Stratasys Ltd., EdenPrairie, MN, USA). Then, 11-cubes in sets of two were printed, each set with a different material for the grid structure: solid bio-compatible MED610 (Stratasys, EdenPrairie, MN, USA) with a Shore hardness of 83-86 (Scale D) and flexible TangoPlus (Stratasys Ltd., EdenPrairie, MN, USA) with rubber-like properties with a Shore hardness of 26-28 (Scale A). SUP706 (Stratasys Ltd., Eden Prairie, MN) was chosen as the support material. The samples were printed with different matrix thicknesses as designed in the CAD software, yielding a specific partial volume contribution C% of the MRI-signal generating material to the voxel.

The spherical tumour models were printed using a different material for the grid structure, TangoPlus, VeroClear and VeroMagenta (Stratasys Ltd.). The general properties of VeroClear and VeroMagenta were similar to those of MED610 but not approved biocompatible. The same support material as for the cubic samples, SUP706, was used.

### 2.4 MR imaging

The cubic samples and spherical models (phantoms) were scanned separately on a 3T PET/MRI system (Siemens Biograph mMR, Siemens Healthineers, Germany) using standard pulse sequences and the mMR spine coil as well as a Tim mMR body coil as utilised in clinical imaging routine. For replicated measurements, the samples were positioned in a similar arrangement with the help of a positioning template. We evaluated MRI visibility for all samples and quantified T1 and T2 relaxation rates over time for the cubic samples. In addition, we performed a Proton Density (PD) MRI image of a *C*% = 100% (MED610 - C100) sample and physiological sodium chloride solution (0.9% NaCl) as a reference to estimate the maximal signal intensity.

All the phantom models were centred in the field of view of the MRI system for the measurements. Images for visualisation were obtained with a variation of a clinically used T1w-2D Spin Echo (SE) pulse sequence with repetition times (TR) of 681 ms, echo time (TE) of 15 ms and a voxel size of VS = 0.9 × 0.9 × 0.9 mm^3^. For T1 relaxation time calculation, a T1-w 2D Steady-State GRE sequence (VS = 1.4 × 1.4 × 5.0 mm^3^) was used, with seven different TRs, ranging from TR = 32 ms to 2050 ms. Further, a T2-w 2D SE pulse sequence (VS = 1.4 × 1.4 × 5.0 mm^3^) was applied to acquire 10 echoes with ΔTE = 20 ms spacing. PD imaging was based on a Turbo SE sequence with a TR of 2900 ms, TE of 14 ms, and a VS = 1.2 × 1.2 × 3 mm^3^. The sequences and parameters used are summarised in Table 1.

**Table 1:** Pulse sequences and their parameters for MR image acquisition. Measurements were performed on a Biograph mMR PET/MRI system using a body coil. HR: High-resolution. Pixel size for T1-w and T2-w images: 1.4 × 1.4 mm^2^.

	**Sequence**	**Flip Angle (°)**	**matrix**	**Pixel size (mm^2^)**	**Slice thickness (mm)**	**TE (ms) [#echoes/echo spacing (ms)]**	**TR (ms)**	**Pixel BW (Hz/px)**	**NEX**	
**T1-w**§	2D Spin Echo	156	256 × 256	0.9 × 0.9	0.9	15	681	155	3	
T1-w*	2D Steady State Gradient-Recalled Echo	90	128 × 128	1.4 × 1.4	5	2	32-2050Δ	375	1	
**T2-w**	2D Spin Echo	180	128 × 128	1.4 × 1.4	5	20-200 [10 / 20]	3000	390	1	
**PD**	2D Turbo Spin Echo		128 × 128	1.2 × 1.2	3	14	2900	200	1	

§ T1-w images for visualisation and mean MRI-signal intensities extraction.

* T1-w images for T1 quantification.

Δ Total TR times acquired were 32, 64, 128, 256, 512, 1028 and 2050 ms.

### 2.5 Visibility analysis

The images for visibility are based on a T1-weighted sequence for clinical applications. We evaluate the change of signal intensity and corresponding contrast arising from the different partial volume contributions C% of the support material. Similarly, the correlation of signal intensity with C% was quantitatively assessed**,** including trend analysis. The mean MRI-signal intensities for the two sets of cubic samples were obtained from circular ROIs (area = 309 mm^2^) placed on the T1-w images. Further, we evaluated the spatially variable MRI signal (gradient) obtained from the three printed copies of the necrotic tumour model through a line profile on the acquired T1-w images**.** For the assessment of the signal intensity, the average signal intensity in the MED610 C100 sample and the physiological sodium chloride solution was measured from a rectangular ROI (area = 400 mm^2^ and 297 mm^2^) placed within the samples, respectively.

### 2.6 Relaxation time calculation

A single circular ROI (area = 309 mm^2^) was placed in a homogeneous region of each set of the T1-w and T2-w images taken from the cubic samples. Mean signal intensity values were obtained from the ROIs. The T1 was estimated according to the progressive saturation method as described, e.g. in [29] by using the generalised fitting equation:

(1) $$s\left(t\right)=a-b*e^{-\frac{\mathrm{TR}}{T1}}$$

Where *a* and *b* are constants related to the spin density and an offset in MRI-signal and *TR* is the repetition time. R2 = 1/T2 rates were obtained by fitting the data to the mono-exponential decay model:

(2) $$s\left(t\right)=\alpha e^{-\frac{\mathrm{TE}}{T2}}+\beta$$

Where α relates to the initial magnetisation, β is an MRI-parameter related offset [30], and TE is the echo time. We excluded the first echo time (TE = 20 ms), as suggested previously [30], for consideration of imperfect rephasing 180° rf-pulses mainly. The fitting analysis was performed in MATLAB R2016a, implementing optimised iterative nonlinear routines.

### 2.7 Relaxation times evaluation

To estimate the variability and stability of the phantoms with regards to the relaxation times, we repeated the T1-w and T2-w measurements seven times over 19 weeks for the two sets of cubic samples. We report mean values and standard deviations (± SD) for the MRI-contrast parameters averaged over the seven time points as a function of C%. To evaluate the long-term stability of the measured T1 and T2 times, we report the results of the relaxation time assessments over the observation period of 19 months for one sample, which showed comparable T1 and T2 times for both evaluated materials.

## 3 Results

### 3.1 Visualisation

**Figure 2:**
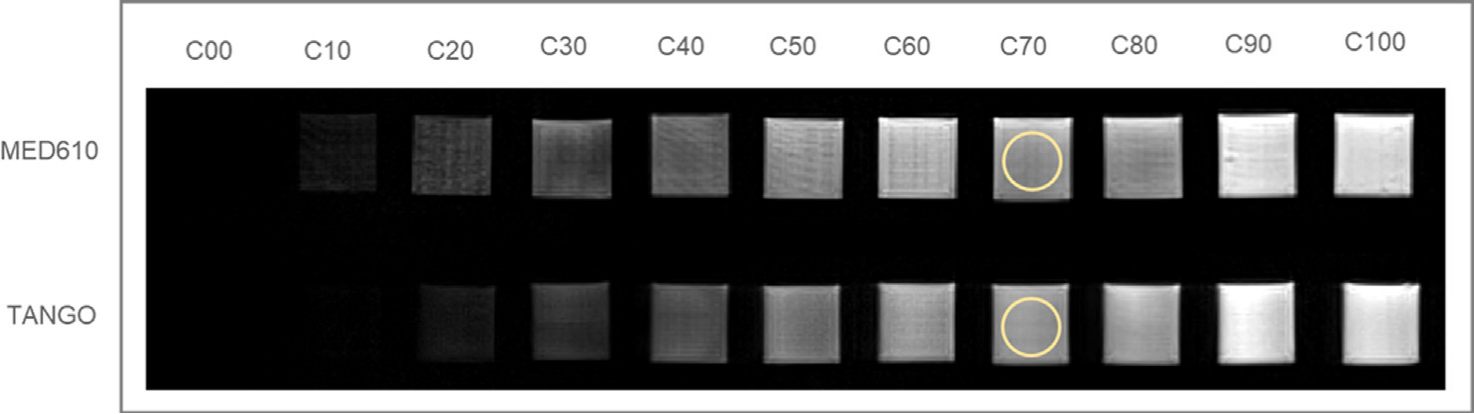
**T1-w MR images of the samples.** Two sets of 11 cubic samples were manufactured using two different materials for the main structure, MED610 (top) and TangoPlus (bottom). Each cubic object contains a different partial volume contribution of MRI-visible support material, ranging from C% = 0% (C00) to C% = 100% (C100). An example of the ROI size and positioning is shown in light yellow on sample C70. The structural artefacts visible in the MED610 samples are expected to arise from a problem with one of the two deposition nozzles used for MED610 in the printing head. This caused a partially imperfect deposition of the MED610 droplets in the printing process.

**Figure 3:**
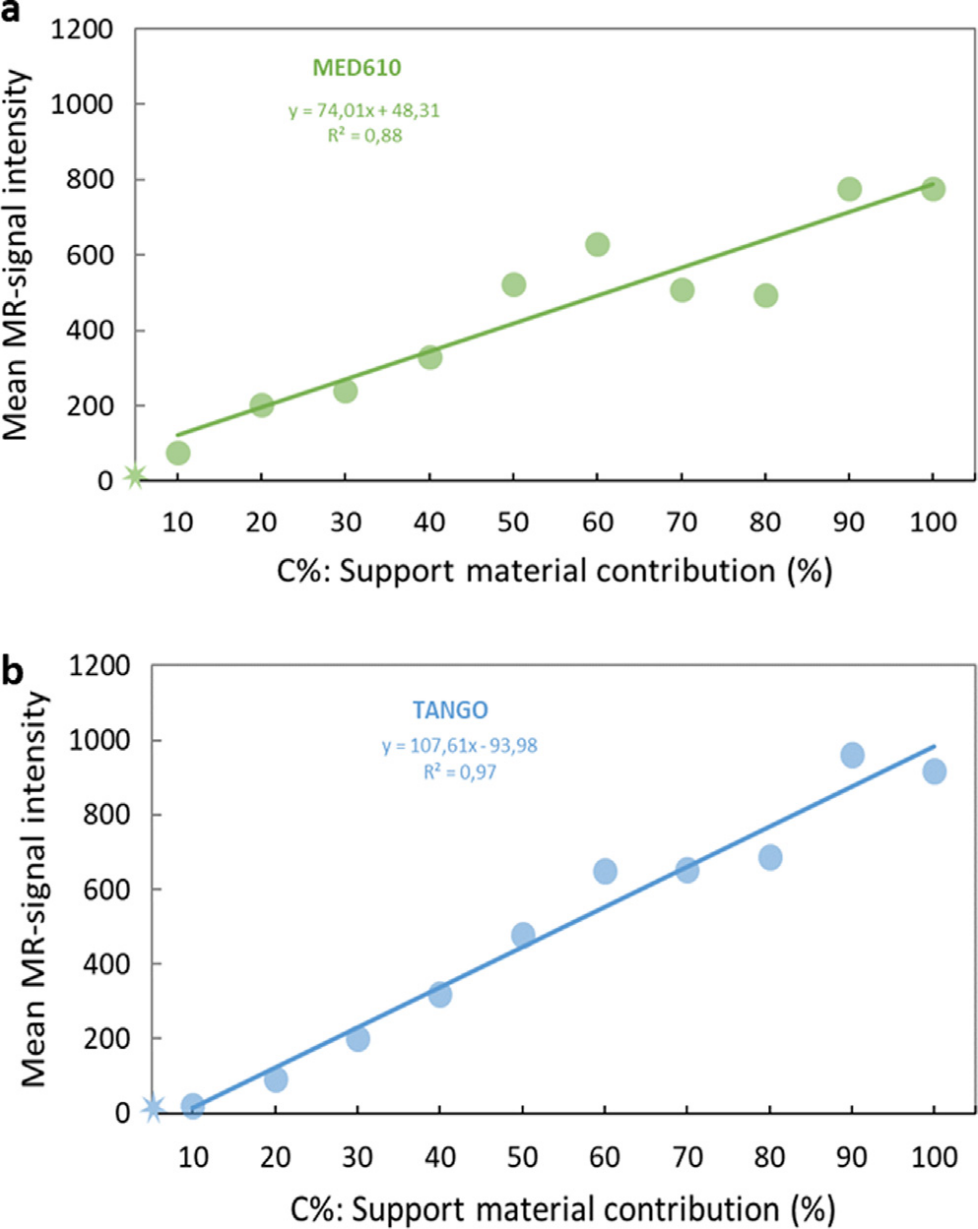
**Mean MRI-signal intensity of the cubic samples as a function of C%.** Mean MRI-signal intensity of the cubic samples as a function of the partial volume contribution of the MRI-visible material for the MED610 (**a**) and the TangoPlus samples (**b**). For both materials, the MRI signal increased with the partial volume contribution of the support material. Note that dropping at C70 and C80 samples may be mainly due to pore size effects and potential supporting material dependence on the two different materials used for the main structure of the phantoms. The asterisk on the plots corresponds to the mean values for the sample C00 (signal intensity = 5.9 units for MED610 and signal intensity = 5.8 units for TangoPlus).

**Figure 4:**
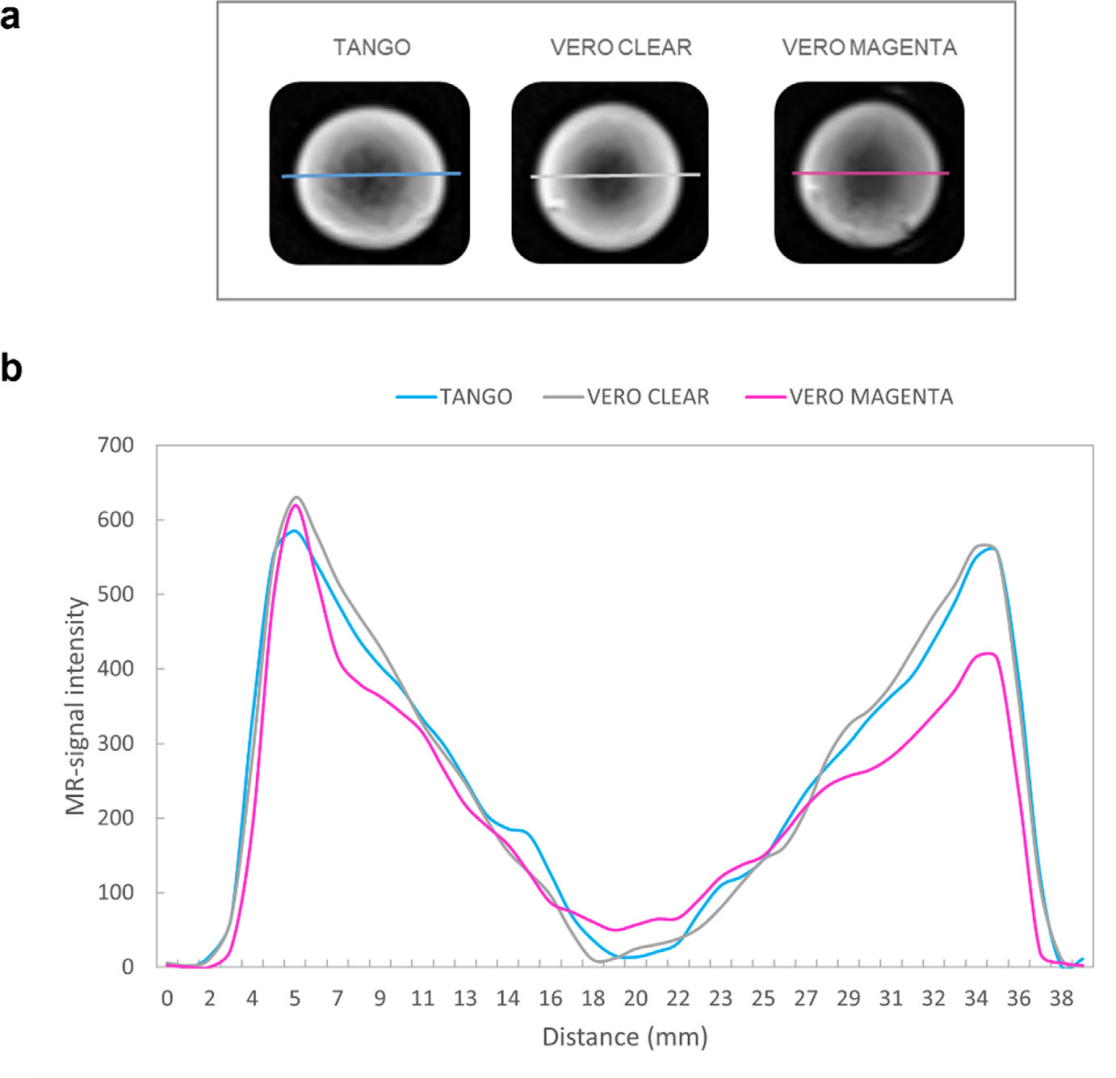
**Images and line profiles of the spherical objects mimicking tumours with a necrotic core.** T1-w MR images of the 38 mm-diameter spherical objects mimicking a necrotic tumour (**a**) and corresponding line profiles (**b**). The initial model was printed with three different materials for the solid structure: TangoPlus^TM^, VeroClear^TM^, and VeroMagenta^TM^, represented by blue, grey and magenta, respectively.

The signal intensities obtained using standard spin- and gradient-echo pulse sequences are shown in Fig. 2. The leftmost images (C00, C10) appear dark due to a null or low partial volume contribution of MRI-visible material. MRI-signal intensities originating from the ROIs placed on the cubic samples and their correlation with the partial volume contribution C% of filling material are presented in Fig. 3. Further, we obtained a smooth and continuous signal gradient with the spherical model, as shown in Fig. 4. The MRI signal in the PD sequence for the C100 sample (pure support material) was 51% (Signal = 844 units) of the signal found for a physiological sodium chloride solution (Signal = 1670 units).

### 3.2 Relaxation times T1 and T2

**Figure 5:**
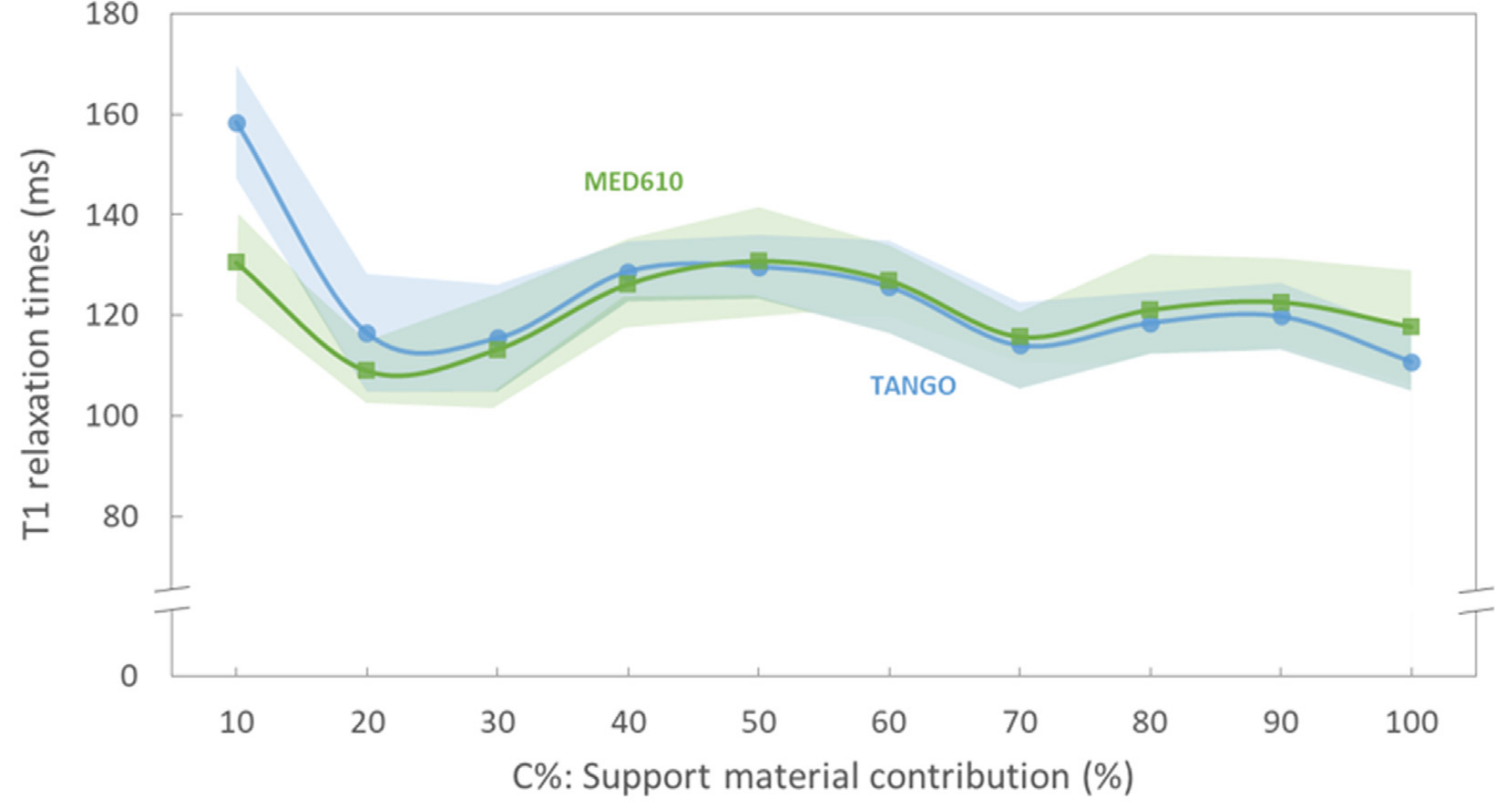
**T1 relaxation times as a function of C% for the different two solid materials.** The lines with markers and shaded areas indicate the measures' average and standard deviation, respectively. The slight differences in T1 values between MED610 and TANGO at 20% < *C* < 100% suggest a low dependence of T1 on partial volume contribution of the filling material. Note the offset in the presentation of the T1-data axis for better visualisation of fluctuations in T1-data.

**Figure 6:**
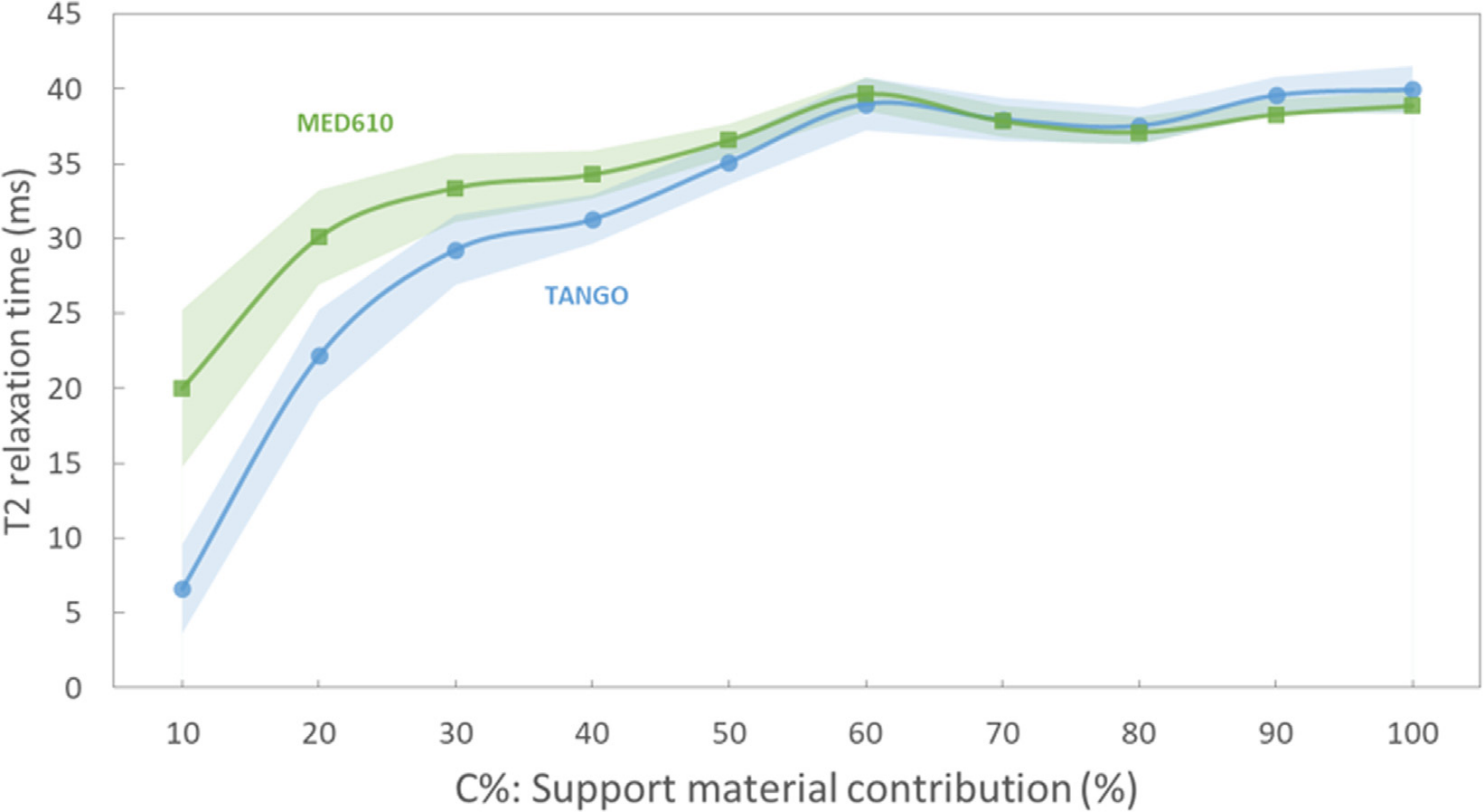
**T2 relaxation times (Mean and SD) as a function of C%.** The T2 times increased with the partial volume contribution of the MRI-visible support material for samples C10 to C60. Note that the material used for the solid structures (MED610 and TangoPlus) and the partial volume contribution of support material C% had less influence on T1 values. Also, T2 variations significantly reduce with higher partial volume contribution of the MRI visible component.

Calculated T1 and T2 relaxation times (Mean±SD) of the samples are summarised in Table 2. For C10 the signal intensity was considerably low, yielding a low signal-to-noise ratio (SNR). Therefore, the high noise in the corresponding images might influence the relaxation times obtained for these samples. For samples C20 to C100, T1 relaxation times ranged between 109 (±6) ms and 131(±11) ms; T2 values ranged between 22 (±3) ms and 40 (±2) ms. In our study, T2 but not T1 relaxation time depended on the partial volume contribution C% of the filling material (Figure 5, Figure 6). T2 values presented a positive correlation with C%. Moreover, T1 values appeared to be independent of the main compartment material; In contrast, T2 values showed higher differences between MED610 and TangoPlus materials for samples with lower C%.

**Table 2:** Relaxation times (mean and SD) for the cube samples averaged for the measurements over 19 weeks (*N* = 7). N.M.: Not measurable.

**Sample**	**T1 (mean±sd) (ms)**		**T2 (mean±sd) (ms)**	
	**MED610**	**TANGO**	**MED610**	**TANGO**
**C00**	N.M	N.M	N.M	N.M
C10*	130.5 ± 8.0	158.4 ± 11.3	20.0 ± 5.3	6.6 ± 3.0
**C20**	109.0 ± 6.0	116.6 ± 11.7	30.1 ± 3.1	22.2 ± 3.1
**C30**	113.3 ± 11.1	115.5 ± 10.6	33.4 ± 2.3	29.3 ± 2.3
**C40**	126.2 ± 9.1	128.8 ± 5.9	34.3 ± 1.6	31.3 ± 1.6
**C50**	130.7 ± 10.8	129.7 ± 6.3	36.6 ± 1.1	35.1 ± 1.5
**C60**	126.9 ± 7.1	125.7 ± 9.2	39.7 ± 1.1	39.0 ± 1.8
**C70**	115.8 ± 4.9	114.1 ± 8.6	37.8 ± 1.0	38.0 ± 1.4
**C80**	121.1 ± 11.0	118.5 ± 6.1	37.1 ± 1.1	37.5 ± 1.2
**C90**	122.7 ± 8.7	119.9 ± 6.6	38.3 ± 1.0	39.6 ± 1.2
**C100**	117.7 ± 11.3	110.9 ± 5.8	38.9 ± 1.0	40.0 ± 1.6

* The values obtained for these samples might encounter high uncertainty due to the low MRI signal provided.

**Figure 7:**
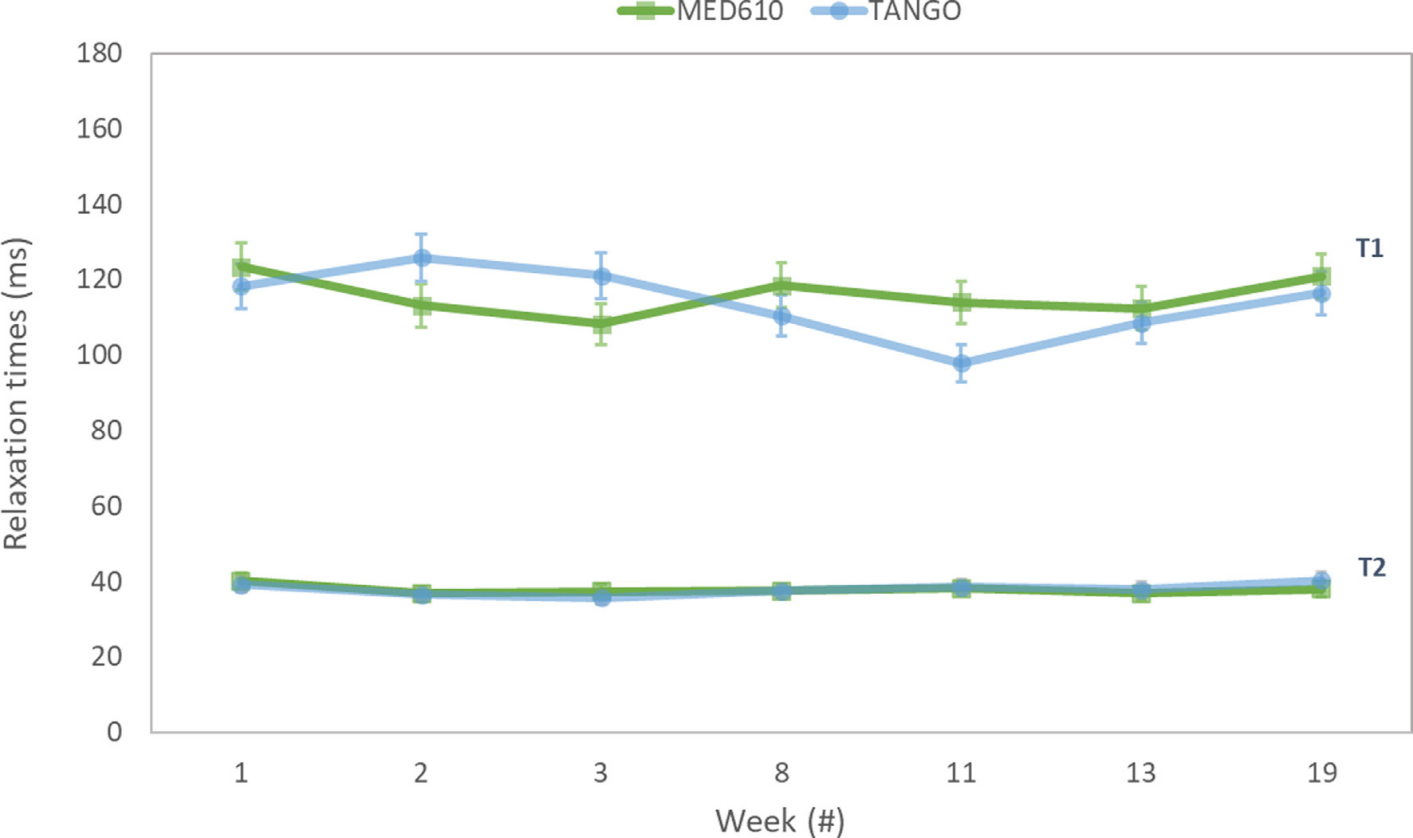
**Stability over time of T1 and T2 relaxation times for sample C70.** Relaxation time values for T1 and T2 for sample C70 over 19 weeks. The T1 and T2 are stable within about 10% for both materials. Error bars indicate a 95% confidence interval for the mean.

From Figure 5, Figure 6, we observed that the samples C70 presented the lowest variability (small SD) between materials for T1 and T2 measurements; we, therefore, selected the two corresponding samples manufactured with MED610 and TANGO to show the long-term stability of the relaxation times. Calculated T1 and T2 times for the two samples over 19 weeks are presented in Fig. 7. In general, the T1 and T2 values of both materials exhibited good long-term stability. However, the variation of T1 and T2 was lower for the MED610 than for TangoPlus samples.

## 4 Discussion

We demonstrated the ability to create objects with adjustable MRI-contrast using standard spin- and gradient-echo pulse sequences in MRI (Fig. 2) without using contrast agents. The signal intensity (and subsequently contrast) could be adjusted solely by varying the partial volume contribution C% of the MR-visible supporting material for AM. The effectiveness of the concept relying purely on the MR-visible spin density contribution to the voxel was proved using a set of phantoms with varying C%, demonstrating the positive and close to linear correlation between the mean MRI signal and the partial volume contribution of the MRI-visible support material (Fig. 3).

Contrary to commonly used multi-compartment phantoms for MRI, the presented technique allows for straightforward creation of phantom objects with varying imaging contrast in substructures without changing printing material using standard double- or multi-head printers for additive manufacturing. The designed objects contain a solid phase and an MRI visible soft supporting material with variable partial volume contributions. The resulting MRI-contrast is based mainly on the number of highly MRI-visible molecules (spin density) of the support material in the voxel; this imaging contrast principle enables the mimicking of tumour heterogeneities (Fig. 4).

For the proposed design, T2 showed a dependence on the partial volume contribution of the MRI-visible support material (Fig. 6). The T2 range of the samples (22 ms–40 ms) is comparable to the reported values for muscle, cartilage and soft tissue such as a liver, at 3.0T [31]. We attribute this variation to a pore size effect within a voxel on T2 originating from the changes in the molecular mobility and potential susceptibility differences between the different printing materials [32]. Therefore, the pore size effect might be used, in principle, for adjusting the T2 of the phantoms.

The printer features a resolution of 600 dpi, allowing a minimal size of a deposited material dot of approx. 0.043 mm size. This enables an adjustment of the inner structure of the printed objects by means of its design and also towards structure sizes far below the selected 1 mm periodicity used in this study. The dimension of the internal cubic grid structure period was chosen to be close to the typical pixel size as an element of resolution in clinical MRI (1 mm^2^). Reducing the size of the inner structure might help to avoid potential artefacts from interference patterns that may arise from specific combinations of basic grid structures similarly sized as voxel sizes (Moire-effect). However, partial volume contribution (spin density) and T2 cannot be adjusted independently and change both with C%, which must be considered when designing these objects.

T1 values are rather short compared to similar reports on relaxation times for body tissues. One limitation of the proposed design is that it does not allow for mimicking the varying contrast with different T1- and T2-weighing sequences. This is because T2 but especially T1 values highly depend on the MRI properties of the support material used. Since the adjustment of the relaxation time could be advantageous for a broader range of applications, adding other components or modifying parameters during the printing process should be considered for subsequent studies. This can also be done in the light of microscopic effects, such as the correlation time and tumble of the molecules, which play an essential role in the macroscopic properties of MRI signal properties [33], [34], [35].

Overall, long-term stability of the T1 and T2 relaxation times was observed (Fig. 7); however, slight differences were found between the two solid materials used for the main structure of the samples. The MED610, the TangoClear^TM^ and the TangoMagenta materials are rigid polymers, while TangoPlus is a flexible polymer. Therefore, external environment conditions may affect the properties of the flexible material and the MRI-signal characteristics of the support material, e.g., oxidation. In our study, small increments in the weight of the samples over time (data not included in this report) were observed for the TangoPlus samples, at around 0.1-0.2% change in weight. No considerable mass variations of the MED610 based samples were observed.

The proposed concept offers MRI-contrast in tissue-mimicking phantoms using the polyjet printing technique for different MRI measurement protocols beyond the simple structural and morphological design of organs and their substructure. We demonstrated a possible mimicking of necrotic tumour heterogeneity; however, slight differences between the images and line profiles obtained from the three replicates of the tumour model were observed. For example, although the same CAD design (the same .stl file) was used for printing, subtle patterns of asymmetric inhomogeneity were seen at some borders of the imaged objects, varying with different printing materials (Fig. 4a). One reason for observing these inhomogeneities is that the proposed principle of partial volume contribution to the pixel relies on pore sizes (of the printed solid matrix) significantly smaller than the voxel in MRI. Otherwise, the pixel intensity varies with the voxel position meeting the solid bar or the soft MRI-visible material. Also, the printing performance for the three different materials might be affected differently by the support material, which might result in slightly different partial volume contributions, especially at very small and very high relative contributions C% (Fig. 5). Further, differences in electromagnetic properties of the printing materials (e.g., magnetic susceptibility, permittivity, conductivity) can lead to inhomogeneous B0 and B1, that can alter MR image intensities. Furthermore, while we used a template for reproducible positioning of the samples throughout all measurements, the use of surface coils with inhomogeneous profiles might have also contributed to signal variability.

The support material used for the manufacturing of the phantoms offered a strong MR-signal intensity that can be easily differentiated by the human eye on a grayscale representation. This implies that the partial volume contribution of the support material to a voxel can be utilised to mimic different hypo- or hyper-intense MRI-appearance (using spin density contribution) in a phantom adapted to the organ or tissue structure of interest. Nevertheless, the support material is intended initially to be removed by flushing liquids after the printing process. Therefore, its utilisation requires caution when not being protected from liquids from outside; it can be achieved by an enclosing shell established by the solid parts of the phantom design.

Finally, the ability to additively manufacture solid objects with controllable MRI contrast seems promising for producing heterogeneous tissue models. It can also be advantageous for various applications, including creating phantoms for specific quality control tests and training in MR-imaging, surgical planning, and MRI-visible needle guides for MRI-guided interventions. Likewise, there is a potential to construct positioning devices for radiotherapy planning based on MRI or combined imaging modalities such as PET/MRI.

## Funding

This project has received funding from the European Union's Horizon 2020 research and innovation programme under the Marie Skłodowska-Curie grant agreement No. 764458. The work reflects only the authors' view, and the Agency is not responsible for any use that may be made of the information it contains.

Additive manufacturing infrastructure for this study was supported by the Austrian Research Promoting Agency (FFG) within the M3dRES project (Grant No. 858060).

## Declaration of Competing Interest

The authors declare the following financial interests/personal relationships which may be considered as potential competing interests: [The Medical University of Vienna has applied for a patent concerning the building technique of solid objects with variable MRI contrast. Ewald Unger and Ivo Rausch are quoted as inventors. The authors declare that the research was conducted without any further commercial or financial relationships that could be construed as a potential conflict of interest.]
